# Massive Open Online Courses-based blended versus face-to-face classroom teaching methods for fundamental nursing course

**DOI:** 10.1097/MD.0000000000024829

**Published:** 2021-03-05

**Authors:** Wenjing Cao, Lin Hu, Xiaoying Li, Xiaoling Li, Chuan Chen, Qianqian Zhang, Shunwang Cao

**Affiliations:** a Nursing School; b School of Public Health; c School of Rehabilitation, Xiang Nan University, Chenzhou; d Department of Laboratory Medicine, The Second Affiliated Hospital of Guangzhou University of Chinese Medicine, Guangzhou, Guangdong Province, China.

**Keywords:** Blended teaching, Massive Open Online Courses, Nursing education, Online learning

## Abstract

An increasing number of studies focus on the effectiveness of Massive Open Online Courses (MOOC)-based blended learning, whereas none have yet studied using it for teaching fundamental nursing skills at an undergraduate level.

To evaluate the effectiveness of MOOC-based blended learning versus face-to-face classroom teaching techniques within the fundamental nursing course at the Faculty of Nursing, University of Xiang Nan, China.

This cluster randomized controlled trial enrolled 181 students and assigned them into either an MOOC-based blended or a face-to-face classroom teaching group, both involving the Fundamental Nursing course for undergraduate nursing students. The analyzed outcomes included test scores, critical thinking ability, and feedback received from the students on the Fundamental Nursing course.

MOOC-based blended techniques versus face-to-face classroom teaching methods demonstrated higher daily performance (*P* = .014), operational performance (*P* = .001), theoretical achievements (*P* < .001), and final grades (*P* < .001) in Fundamental Nursing.

Moreover, the mean change in the participants’ critical thinking ability items between groups were, mostly, statistically significant. The items focusing on the feedback from the students demonstrated significant differences between the groups in terms of their satisfaction with the teaching they received (*P* < .001) and the overall learning effects (*P* = .030).

This study confirmed that receiving MOOC-based blended learning was superior when compared against face-to-face classroom teaching techniques for learning within the Fundamental Nursing course.

## 1 Introduction

Due to the development of the internet, major changes in peoples’ lives have been produced, with one of the most important being the applications of web-based learning. Currently, there is an increasing drive, internationally, aimed at incorporating web-based learning into higher education. Massive Open Online Courses (MOOCs), as a new online learning approach, has been an intense focus of educational research within a short time period.^[1–5]^ Nowadays, a wide variety of subjects utilize MOOCs.^[6,7]^ They attract larger numbers of learners, worldwide, because of the following strengths:

(1) delivering academic content with unlimited and open registrations;
(2) giving students the opportunity to share their knowledge and expertise with one another and to manage their own learning, enhancing interactions (not only among students, but also between them and their instructors); and (
(3) conducted appropriately, it is a valuable tool in spreading subject knowledge in a small time period at low costs. However, many experts believe MOOCs could not possibly, or are not desirable to, fully replace conventional, face-to-face classroom teaching techniques, despite the fact that they are a growing force in higher education.^[6]^

There are increasing debates on how both traditional and asynchronous or synchronous e-learning can be combined for effective teaching. Blended learning has come into being and risen to greater popularity over the last few years.

Blended learning is a positive application of MOOCs management platform, defined as the combination of traditional face-to-face learning techniques, and a number of pedagogic approaches supported by information and communication technology.^[8,9]^ It is now widely used in education as it has the potential to achieve desired learning outcomes. It has been driven in higher education to unprecedented levels, with a new focus on interactive, student- centered learning.^[10]^ Various studies have aimed to evaluate the effect of blended learning for teaching pharmacology and other medical subjects.^[11,12]^ However, studies showing whether MOOC-based blended teaching was superior than face-to-face classroom teaching for nursing education remained limited and inconclusive.^[9]^

Almost all nursing education is teacher-oriented and conventionally focused on lecture-based strategies in order to disseminate factual knowledge. However, as health care is becoming increasingly sophisticated, countries are facing challenges around their graying populations with chronic diseases, and a greater emphasis is placed on higher quality practices. It is, therefore, not possible to cultivate capable nurses using traditional teacher-centered lecturing. Therefore, it is of integral importance for countries to move towards more learner-centered teaching strategies to strengthen and develop their students’ test scores and critical thinking abilities, as well as improve the overall feedback received from students regarding the Fundamental Nursing course. As one of the most important aspects within nursing education, the Fundamental Nursing course plays an important role in cultivating capable nurses. Creating an appropriate training environment and improving the current methods in this course is a priority as a result. This study was conducted to assess the effectiveness of MOOC-based blended learning versus face-to-face classroom teaching in the Fundamental Nursing course among university students.

## 2 Methods

### 2.1 Study design, participants, setting, and measures

This study was conducted using a prospective cluster randomized controlled design aimed at examining the effects of MOOC-based blended learning versus face-to-face classroom teaching methods on the test scores, critical thinking abilities, and feedback of third year nursing undergraduate students. The test scores were considered as the primary endpoint, and the calculation of the sample size based on α and β values of 0.05 and 0.20, respectively. The enrolled population for this study included 181 nursing students in the third year of a four-year undergraduate program, who had attended the Fundamental Nursing II course in the School of Nursing, Xiang Nan University, Hunan province, China. There were four classes of third-year students in this nursing school. Since all of the classes had already undergone Fundamental Nursing I, and had passed the subsequent exam, their final scores on the Fundamental Nursing I Test were compared in order to verify that they had similar knowledge in that subject. We randomly selected two classes, who were then randomly assigned to 1 of 2 groups: the MOOC-based blended teaching group (n = 91), and the conventional, face-to-face classroom teaching group (n = 90). The two instructional strategies (MOOC-based blended and conventional, face-to-face classroom teaching methods) were the independent variables, with the dependent variables being the test scores, critical thinking abilities, and overall feedback of students. It is worth mentioning that the experiment was conducted during the 2017 autumn semester.

### 2.2 Ethical statements

The study does not involve patients, patient tissue or patient data, so ethical review of clinical studies is not applicable.

### 2.3 Study Instruments

The instruments for this study included a demographic questionnaire, test scores, critical thinking abilities, and feedback all received from students. The demographic questionnaire covers the following three items: sex, age, and the final scores on the Fundamental Nursing I Test. The scores in final grade consisted of daily performance (10%) + operating performance (20%) + theoretical achievements (70%). The definition of daily performance including performance in class and lab reports, operating performance was defined as the objective structural assessment of nursing operation in clinical, and theoretical achievements was considered as knowledge of the inspection. The Chinese version of the Critical Thinking Disposition Inventory was employed in order to measure participants’ critical thinking abilities.^[13]^ This tool consists of 70 items, each scored on a 6-point Likert scale, from 1 “not at all” to 6 “always.” Among the 70 items, 10 focus on truth seeking, 10 survey for open mindedness, 10 measure decision-making to determine analytical abilities, 10 measure systemic capacity, 10 measure critical thinking self-confidence, 10 focus on inquisitiveness, and the remaining 10 cover cognitive maturity. A prior study proved that the Critical Thinking Disposition Inventory has high content and construct validity.^[13]^

The scale measuring the feedback received from students comprised five parts with 18 items and was designed exclusively for this study. All of the items were developed according to the standardized teaching feedback style of our university. Subsequently, two seasoned nursing specialists (each with >10 years of experience) and one educational expert provided feedback on, and verified the suitability of, the items and the scale's overall content validity. A blank space was placed around each item to write opinions, as obtained from the experts, regarding any modifications needed to verify the scale's content validity. Each item's validity index ranged from 0.8 to 1.0. The scale included four items on students’ overall satisfaction with the teaching style, three on learning effects, four on self-study status, four on operational practice, and three on teamwork:

(1) the satisfaction with teaching items assessed students’ happiness with the teaching methodology, existing learning resources, quality of taught content, and teacher-student interactions;
(2) the learning effect items assessed whether there were clear learning goals, if they kept up with the learning progression, and mastery of the knowledge points;
(3) the self-study status items assessed attitude, learning enthusiasm, awareness, and ability;
(4) the operational practice items assessed abilities in assessing illnesses accurately, mastering operations skillfully, performing various operations correctly, and giving correct health guidance; and
(5) the teamwork items assessed group awareness, abilities to work with others, and communication abilities.

Each item was scored on a three-point Likert scale, ranging from 0 “very unsatisfactory” to 2 “very satisfied.” Cronbach α was an average of 0.94 for all items, and specifically 0.92 for the satisfaction with teaching style items, 0.93 for the learning effect items, 0.91 for the self-study status items, 0.93 for the operational practice items, and 0.91 for the teamwork items.

### 2.4 Training curriculum

The Fundamental Nursing II course is given to third-year nursing students, with the content created utilizing established principles of curriculum development. It is composed of 22 hours of theoretical courses, and 26 hours of practical courses covering injection methods, disease observations, pain care, and hospice care. We selected these highly prevalent contents for the intervention. We designed course plans, lesson plans, timetables, and tests for the Fundamental Nursing II course, for both conventional and MOOC-based blended teaching strategies in advance. Learning objectives, course lecture slides, course materials, homework, and operation assessment items were the same for both groups and were taught by the same teachers. Structured live group activities and case discussions were included in the two groups in order to complement lectures.

The conventional, face-to-face classroom teaching group underwent learning involving conventional instructions in full lectures. Each class was conducted according to the curriculum schedule: homework and tests were completed, relevant pre-class materials were assigned by the teacher before classes, the pre-class situation was analyzed by a questionnaire method, and new knowledge was taught (combined with clinical cases) using traditional teaching methods.

In the MOOC-based blended teaching group, lectures would be recorded by video and made available to students on their computers via the Internet, using the University's MOOC platform, before classes. Afterwards, both teachers and students gathered for discussion, guidance, and reflection on the content of the recorded lectures in group sessions. The Fundamental Nursing II course based on MOOC was created for the academic semester of May to December 2016. The teaching process of the MOOC-based blended teaching was conducted as shown in Table 1.

**Table 1:** The process of MOOC-based blended teaching.

Link	Teachers’ activities	Students’ activities
Link before class	The platform issues learning notice and requirements, conducts studies, teaches video, raises questions; The teacher designs the classroom teaching content according to the feedback of students’ learning survey form.	Students can watch video within the specified time limit, and complete the preliminary study independently. Students can prepare the problem book, record the problems at any time before class, which was received examination by an independent teacher. After learning the teaching of video, students should fill in the study survey form in time and record their understanding of video (percentage) and problems encountered. Before class, the electronic study survey form will be collected and sent to the teacher by the study committee.
Link in class	The teacher is responsible for prompt and answer questions. The basic knowledge is no longer repeated, and the flipped teaching forms such as case application, scenario demonstration and student demonstration are used to carry out in-depth discussion on the key and difficult points. To solve common and individual problems of students and inspire students to solve independently; Teacher comments or make a summary of the course content.	Learning group report: students work in groups to make study reports in class and set challenge sessions. Other students can challenge the content of the report. If the group cannot answer the questions, the group will score 0.Group discussion: students discuss problems they encounter in class.
Link after class	Assign homework, pay attention to students’ questions in bulletin board system area of MOOC platform and interact with students in a timely manner.	Finish the homework in groups.Consult relevant literature and solve problems independently;You are free to choose a subject related to the course and complete a book report.

### 2.5 Statistical analysis

The study sample was analyzed via descriptive statistical analyses, and the baseline characteristics of the students between groups are presented as mean (standard deviation)/median (quartile), and event (percentile) for continuous and categorical data. Both Student *t*- and Mann–Whitney *U*-tests were employed in order to assess differences between the groups in terms of continuous data based on overall normality. A chi-square test was employed to evaluate the differences between groups in terms of their categorical data. Moreover, the mean differences were analyzed in order to evaluate the changes in critical thinking abilities, with the differences between groups being assessed by a Student *t*- or Mann–Whitney *U*-test. Statistical significance was considered at *P* < .05. All statistical analyses were performed using SPSS 21.0 (SPSS Inc, Release 21.0).

## 3 Results

### 3.1 Characteristics of enrolled sample

A total of 181 participants (24 male and 157 female) participated in the study, with all demographic data collected. Table 2 provides the demographic characteristics of the study participants within each group. There were no significant differences between MOOC-based blended and face-to-face classroom teaching groups in terms of sex (*P* = .168), age (*P* = .116), or final scores on the Fundamental Nursing I Test (*P* = .964).

**Table 2:** Demographic characteristics of study participants (N = 181).

Variables	MOOC-based blended teaching group (n = 91)	Face-to-face classroom teaching group (n = 90)	*P* value
Sex, n (%)			
Female	78 (85.70)	79 (87.80)	.168^∗^
Male	13 (14.30)	11 (12.20)	
Age, yr (median and quartile)	20.00 (19.50∼21.00)	21.00 (19.50∼21.00)	.116^†^
Final scores on Fundamental Nursing I Test (mean and standard deviation)	78.30 ± 8.35	78.23 ± 10.38	.964^‡^

### 3.2 Test scores

Table 3 describes the results for the daily performance, operational performance, theoretical achievements, and final grades between the 2 groups. The final grades at the end of the course were a sum of the students’ activity throughout the term: daily performance (10% of final grade), operational performance (20% of final grade), and the final practical exam (70% of final grade). Overall, students in the MOOC-based blended teaching group were associated with higher scores for operational performance (*P* = .001), theoretical achievements (*P* < .001), and final grades (*P* < .001) compared to those in the face-to-face classroom group. However, the daily performance in MOOC-based blended teaching group was significantly lower than face-to-face classroom group (*P* = .014).

**Table 3:** Comparing scores between the MOOC-based blended teaching and conventional, face-to-face classroom teaching groups.

Variables	MOOC-based blended teaching group (n = 91)	Face-to-face classroom teaching group (n = 90)	*P* value
Daily performance	87.00 (80.00∼94.00)	91.00 (85.00∼94.00)	.014^∗^
Operating performance	80.00 (74.00∼85.00)	76.00 (70.00∼80.00)	.001^∗^
Theoretical achievements	87.00 (80.00∼93.00)	80.00 (72.75∼87.00)	<.001^∗^
Final grade	85.20 (79.60∼90.40)	80.00 (74.63∼85.85)	<.001^∗^

### 3.3 Critical thinking ability

The characteristics of the critical thinking abilities between the MOOC-based blended and face-to-face classroom teaching groups are shown in Table 4. Overall, at baseline, the items forming critical thinking ability - including truth seeking (*P* = .706), open mindedness (*P* = 0.892), analytical ability (*P* = 0.706), systemic capacity (*P* = .844), critical thinking self-confidence (P = 0.941), inquisitiveness (*P* = .143), and cognitive maturity (*P* = 0.067) were not statistically significant between groups. However, the items forming critical thinking ability, including truth seeking (*P* < .001), analytical ability (*P* < 0.001), critical thinking self-confidence (*P* < 0.001), inquisitiveness (*P* < .001), and cognitive maturity (*P* < 0.001), following the intervention in the MOOC-based blended group were significantly improved than those in face-to-face classroom teaching group. However, the improvement in open mindedness (*P* < .001), and systemic capacity (*P* < .001) in MOOC-based blended group were inferior than those in face-to-face classroom teaching group. Finally, there was no significant difference between MOOC-based blended and face-to-face classroom teaching groups for the improvement in cognitive maturity (*P* = .473).

**Table 4:** Critical thinking abilities across students randomized to MOOC-based blended and face-to-face classroom teaching.

Items	Group	MOOC-based blended teaching group (n = 91)	Face-to-face classroom teaching group (n = 90)	*P value*
Truth seeking	Before	25.00 (25.00∼26.00)	25.00 (24.25∼27.00)	.706
	After	38.00 (38.00∼40.00)	34.00 (32.00∼37.00)	<.001
	Mean change	13.00 (13.00∼15.00)	9.00 (7.00∼11.00)	<.001
Open mindedness	Before	27.00 (27.00∼28.00)	27.00 (25.25∼29.00)	.892
	After	28.00 (25.00∼31.00)	30.50 (29.00∼33.00)	<.001
	Mean change	0.00 (-2.00∼4.00)	3.00 (0.00∼6.25)	<.001
Analytical ability	Before	36.00 (35.00∼37.00)	36.00 (34.25∼3725)	.706
	After	37.00 (36.00∼38.00)	27.00 (24.00∼27.00)	<.001
	Mean change	1.00 (0.00∼2.00)	-9.50 (-12.00∼-8.75)	<.001
Systemic capacity	Before	33.00 (32.00∼35.00)	33.00 (31.25∼36.50)	.844
	After	32.00 (30.00∼33.00)	38.00 (34.75∼38.00)	<.001
	Mean change	-2.00 (-5.00∼0.00)	4.00 (1.00∼6.00)	<.001
Critical thinking self-confidence	Before	27.00 (25.00∼28.00)	26.00 (26.00∼27.00)	.941
	After	43.00 (42.00∼44.00)	27.00 (27.00∼28.00)	<.001
	Mean change	17.00 (14.00∼18.00)	1.00 (0.00∼2.00)	<.001
Inquisitiveness	Before	22.00 (20.00∼23.00)	22.00 (20.25∼24.75)	.143
	After	38.00 (37.00∼38.00)	26.00 (25.00∼29.00)	<.001
	Mean change	16.00 (14.00∼18.00)	4.00 (1.75∼7.00)	<.001
Cognitive maturity	Before	28.00 (28.00∼28.00)	28.00 (27.00∼28.00)	.067
	After	32.00 (31.00∼32.00)	31.00 (31.00∼31.75)	<.001
	Mean change	4.00 (3.00∼6.00)	3.00 (3.00∼4.75)	.473

### 3.4 Feedback from students

The feedback received from the students following the intervention between groups is presented in Table 5. There were significant differences between the groups for overall satisfaction with the teaching methods (*P* < .001) and learning effects (*P* = .030), whereas there were no significant differences for the scores of self-study status (*P* = .065), operational practice (*P* = .073), and teamwork (*P* = .796).

**Table 5:** The differences in feedback received from students between the MOOC-based blended and face-to-face classroom teaching groups.

Variable	MOOC-based blended teaching group (n = 91)	Face-to-face classroom teaching group (n = 90)	*P* value
Satisfaction with teaching	6.00 (5.00∼7.00)	5.00 (4.00∼6.00)	<.001^∗^
Learning effect	5.00 (4.00∼5.00)	4.00 (3.00∼5.00)	.030^∗^
Self-study status	6.00 (5.00∼7.00)	6.00 (4.00∼6.00)	.065
Practice operations	6.00 (5.00∼6.00)	5.00 (4.00∼6.00)	.073
Teamwork	4.00 (4.00∼5.00)	4.00 (3.00∼5.00)	.796

## 4 Discussion

The main purpose of this experiment was to investigate the effects of MOOC-based blended versus face-to-face classroom teaching strategies on test scores, critical thinking abilities, and feedback of students enrolled in the Fundamental Nursing course. The results of the study revealed that students in the MOOC-based blended teaching group were associated with lower daily performance, but higher operational performance, theoretical achievements, and final test scores. Moreover, the mean changes in critical thinking ability items significantly changed in the MOOC-based blended teaching group compared to those in the face-to-face classroom teaching group, excluding cognitive maturity. Finally, students in the MOOC-based blended teaching group were associated with higher satisfaction with teaching methods and learning effects compared against those in the face-to-face classroom teaching group, whereas no significant differences for self-study status, operational practice, and teamwork were observed.

The current study suggests that MOOC-based blended teaching strategies are associated with significantly improved final grades, specifically in terms of operational performance and theoretical achievements, whereas the daily performance in the MOOC-based blended teaching group was lower than those of the face-to-face classroom teaching group. These results are consistent with previous studies,^[14–18]^ and could explained by:

(1) MOOC-based blended learning offers increased flexibility in transferring new knowledge and skills;
(2) MOOC platforms allow students to gain access to contents in a variety of different formats and to fill in the gaps in their knowledge;
(3) students in the MOOC-based blended teaching group spent more preparation time outside of the classroom than the students in the traditional face-to-face teaching group; and
(4) the MOOC students were able to foster learning and understanding of the content materials more quickly and correctly through discussions, guidance, and reflections on the content of the recorded lectures in group sessions after self-studying the basic knowledge.^[8,17,19]^

However, another study did not find any significant difference between groups for students’ knowledge and skills.^[11]^ A potential reason for this could be that some students, who are accustomed to a traditional method, may resist spending more time in self-directed learning.^[20]^ Moreover, differing techniques, subjects, qualities, processes, and lengths of interventions can bring about different educational effects. Therefore, it is important to recognize that, only if blended teaching was highly structured (with a focus on higher quality methods and utilizing tutorials) would it be beneficial over purely traditional learning.

The results of this study suggested that MOOC-based blended teaching strategies were associated with significant improvements in most items of critical thinking ability when compared against face-to-face classroom teaching strategies. This result is corroborated by previous studies demonstrating the ability of blended learning in facilitating the “flipped classroom” model.^[21–23]^ A possible explanation may be that we planned the MOOC-based blended teaching carefully and focused on delivering higher levels of thinking skills, such as synthesis or evaluation of knowledge instead of just covering the basics. Moreover, we tried our utmost to stimulate active and self-directed learning via providing different resources that could be transited to constructivist teaching. This is in agreement with the work of Boelens et al.^[24]^ Moreover, students might potentially engage more with a constructivist environment by analyzing, interpreting, and discussing during the learning process, keeping them active. Several reasons could explain these significant changes:

(1) students in the MOOC-based blended teaching group are closer to the practical reality of nursing, which increases the abilities of problems solving and self-directed learning;
(2) the virtual process of MOOC-based blended teaching strategies could motivate students to answer critical thinking questions; and
(3) students receiving MOOC-based blended teaching methods could develop a mind map of a complete holistic nursing process, which is associated with significant improvements in critical thinking skills.

In addition, this study indicates that MOOC-based blended learners expressed more positive feedback with respect to the teaching situation, including greater satisfaction with both the teaching and learning effects. It also offers potential improvements in students’ self-study status, operational practice, and teamwork — more so than face-to-face classroom strategies. These results are in line with the findings of other studies, which showed that student feedback concerning the blended learning approach was predominantly positive.^[25–27]^ However, it must be noted that the improvements in student satisfaction might be due to a novelty effect and is an area for continued exploration. Moreover, the MOOC-based blended approach was not appreciated by all students in a previous study.^[28]^ This result could be due to them losing opportunities for student/teacher interactions, the process of interventions, and online connection issues. Furthermore, students had been exposed only to traditional learning formats prior to this study, and, as a result, they may not have developed more self-directedness in their learning, which may have influenced their satisfaction ratings.^[29]^

Although designed to build upon previous literature, this study still had several limitations. The study examined groups of students in two extreme learning environments: a group of students exposed to exclusively face-to-face classroom teaching methods with no MOOC, and a group of students who exclusively experienced the MOOC-based blended teaching method. However, this study did not compare a group of students exposed to face-to-face classroom teaching methods with minimal use of MOOCs, with the group who underwent the MOOC-based blended teaching method. This could potentially affect students’ overall performance in a negative way and, thus, we did not include this in the present study. Moreover, this study was conducted in a single school, which limits generalizability, and therefore provides no information about the scalability of blended learning. This will need to be explored in further studies. Furthermore, the assignment of the classes to each approach was purely randomized, and the students did not have a choice in their participation in the study, as the Fundamental Nursing course is obligatory, and the official programs of the universities could not be changed. Finally, students included in this study have already completed Fundamental Nursing I course, and the conclusion of this study was biased owing to the study did not show the effect of different teaching methods on the students who are first time being exposed to a new course.

Our study found that the effectiveness of the MOOC-based blended teaching methods is an improvement on the face-to-face classroom teaching techniques utilized in the Fundamental Nursing course, in terms of test scores, critical thinking abilities, and feedback received from students. Nursing teachers could use MOOC-based blended teaching appropriately and adopt reasonable suggestions from the students for further improvement in such aspects as the course form, content design, and evaluation.

## Acknowledgments

The authors thank all of the teachers from Basic Nursing Teaching and Research Office, School of Nursing, Xiang Nan University for building Fundamental nursing MOOC course.

## Author contributions

**Conceptualization:** Wenjing Cao, Xiaoling Li.

**Data curation:** Wenjing Cao, Xiaoying Li, Chuan Chen, Qianqian Zhang.

**Formal analysis:** Lin Hu.

**Writing – original draft:** Wenjing Cao, Xiaoying Li.

**Writing – review & editing:** Wenjing Cao, Shunwang Cao.
